# Long-Term Cure of Primary Hyperparathyroidism After Scan-Directed Parathyroidectomy: Outcomes from a UK Endocrine Surgery Unit

**DOI:** 10.1007/s00268-022-06543-8

**Published:** 2022-06-07

**Authors:** Stan Sidhu

**Affiliations:** Endocrine Surgery Unit, Royal North Shore Hospital and the University of Sydney, St Leonards, NSW Australia

In this issue, Patel and Mihai describe their long-term outcomes in a large cohort of patients undergoing minimally invasive parathyroidectomy (MIP) for a single parathyroid adenoma, after concordant dual localisation with Sestamibi scintigraphy and ultrasound scan [1]. They have demonstrated durable long-term outcomes utilising this approach in expert hands, without the use of intraoperative PTH measurement (IOPTH), reporting a very low recurrence rate of 3.85% in a cohort of 390 patients, with a median follow-up time of 78 months. This large series contributes valuable evidence that supports scan-directed MIP as an effective and valid operative approach.

Endocrine surgery is in a constant state of flux, with controversies regarding the optimal technique for thyroid surgery, the appropriate extent of thyroidectomy; hemi versus total thyroidectomy, with or without prophylactic central node dissection for papillary thyroid cancer, or the relative benefits of anterior versus posterior endoscopic approaches for minimally invasive adrenal surgery. The appropriate operative approach for primary hyperparathyroidism is the subject of similar controversy. In many centres around the world, the use of IOPTH is increasing. Although it may provide helpful guidance in a non-localised patient with equivocal intraoperative findings, or in patients undergoing reoperation after failure to cure, its routine use in well-localised patients undergoing MIP can be reasonably avoided. It is an inefficient and expensive exercise (unless undertaken in a dedicated high volume parathyroidectomy theatre), and based on the number needed to treat calculated with Patel and Mihai’s results, the cost–benefit equation for IOPTH use would be difficult to balance. However, it needs to be stressed that careful patient selection must be informed by reliable concordant functional and anatomical preoperative imaging.

Patel and Mihai’s results mirror the Australian experience of the University of Sydney endocrine surgery unit and the Monash endocrine surgery unit [2, 3], who over the last two decades have practiced according to a similar treatment algorithm. Furthermore, in a recent large series of 609 patients, the Hammersmith group demonstrated equivalent cure rates of 97.9% for dual localised patients whether they underwent unilateral or bilateral neck exploration, as well as no difference in cure rates when assessing the efficacy of the use of IOPTH [4]. Taken together, these concordant international multicentre results provide an evidence base to support a sensible and practical approach to MIP, avoiding the routine use of IOPTH or frozen section, while maintaining excellent surgical outcomes. The authors should be congratulated on their results and for providing us with their long-term follow-up data.
